# Cisatracurium besilate enhances the TRAIL-induced apoptosis of gastric cancer cells via p53 signaling

**DOI:** 10.1080/21655979.2021.2009318

**Published:** 2021-11-30

**Authors:** Qiang Zhou, Jianxia Yuan, Yi Liu, Yayun Wu

**Affiliations:** a Department of Anesthesiology, Jin Yin-tan Hospital, Wuhan, Hubei, China; b Department of Surgery, Wuhan Institute for Tuberculosis Control, Wuhan, Hubei, China; c Outpatient Department, Langli Aesthetic Surgery Clinic, Xi’an, Shanxi, China; d Department of Endoscopy, The Second People’s Hospital of Shanxi Province, Xi’an, Shanxi, China

**Keywords:** Cisatracurium besilate, gastric cancer, TNF-related apoptosis-inducing ligand, apoptosis

## Abstract

Cisatracurium besilate is the most commonly used non-depolarizing muscle relaxant in general anesthesia and in intensive care units. Studies have indicated that the proliferation of gastric cancer (GC) cells can be restrained by cisatracurium besilate. The present study aimed to investigate the mechanism underlying the role of cisatracurium besilate in TNF-related apoptosis-inducing ligand (TRAIL)-induced GC. The AGS cell line was exposed to cisatracurium besilate, and then cell viability, colony formation and apoptosis were assessed by performing Cell Counting Kit-8, colony formation, TUNEL and Western blot assays, respectively. Furthermore, the expression levels of p53 and p53 upregulated modulator of apoptosis (PUMA) were measured by Western blotting to determine the effect of cisatracurium besilate on p53/PUMA signaling. After co-treatment with p53 inhibitor, cisatracurium besilate and pifithrin-α/TRAIL, cell apoptosis was detected. Finally, cisatracurium besilate and pifithrin-α were used to co-treat TRAIL-induced AGS cells followed by apoptosis detection. Cisatracurium besilate treatment restrained the proliferation and promoted the apoptosis of AGS cells. Cisatracurium besilate also promoted the expression of p53 and PUMA in AGS cells. Furthermore, TRAIL induced the apoptosis of AGS cells, which was aggravated by cisatracurium besilate treatment. However, pifithrin-α reversed the synergistic effects of cisatracurium besilate and TRAIL on the activities of AGS cells. Therefore, the present study suggested that cisatracurium besilate enhanced the TRAIL-induced apoptosis of GC cells via p53 signaling, and the synergistic effects of cisatracurium besilate and TRAIL may achieve maximal therapeutic efficacy in GC management.

## Introduction

Gastric cancer (GC) is the fourth leading cause of cancer-related mortality in men and women worldwide [1]. The incidence of GC is higher among individuals with a lower socioeconomic status, whereas its incidence has declined among individuals with a higher socioeconomic status in recent decades [2]. GC is a multistep and multifactorial process. Despite advances in diagnostic studies and therapeutic options for GC in the past decades, there has been no marked improvement in outcome. Currently, it is recognized by translational reports that operative nursing intervention for patients undergoing GC surgery is of great value for postoperative rehabilitation and improving the prognosis of GC [3]. In contrast to routine postoperative surgical care, which can only slightly improve the quality of life of patients with GC, comprehensive operative nursing can effectively reduce stress responses and improve the psychological state of the patients [4].

**Figure 1.**
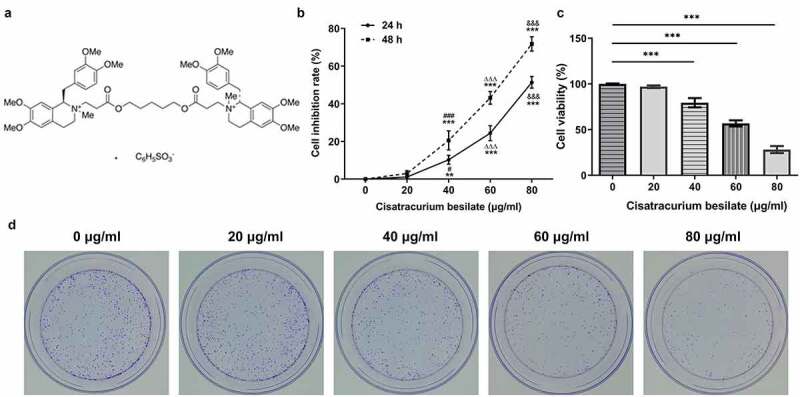
Cisatracurium besilate restrains the proliferation of AGS cells. (a) Chemical structure of cisatracurium besilate. (b) Inhibition rate of cisatracurium besilate-treated AGS cells. **P < 0.01 and ***P < 0.001 vs. 0 μg/ml; ^###^P < 0.001 vs. 20 μg/ml; ^ΔΔΔ^P<0.001 vs. 40 μg/ml; ^&&&^P < 0.001 vs. 60 μg/ml. AGS cell (c) viability and (d) colony formation following treatment with cisatracurium besilate. ***P < 0.001

Cisatracurium besilate (Figure 1a) is the most commonly used non-depolarizing muscle relaxant in general anesthesia and in intensive care units, displaying a lower propensity to trigger adverse cardiovascular effects and no accumulation *in vivo* following clinical use compared with other non-depolarizing drugs [5,6]. However, its adverse effects were suggested to be enhanced by certain congenital heart diseases associated with abnormal hemodynamics [7]. The majority of cisatracurium besilate undergoes Hofmann degradation, which relies on pH and temperature in plasma and tissues [8]. It was previously indicated that the proliferation of GC cells can be restrained by cisatracurium besilate [9]; however, whether cisatracurium besilate exerts the same effect on the apoptosis of these cells remains unexplored. Studies have suggested that cisatracurium besilate can activate p53 signaling to inhibit the proliferation and promote the apoptosis of colorectal cancer cells, and inhibit angiogenesis [10–12]. Moreover, cisatracurium besilate can activate the expression of p53 signaling in esophageal squamous cell carcinoma cells.

However, whether cisatracurium besilate participates in the apoptosis of GC cells via p53 signaling is not completely understood. Therefore, the present study investigated the mechanism underlying the role of cisatracurium besilate in TNF-related apoptosis-inducing ligand (TRAIL)-induced GC.

## Materials and methods

### Cell culture

The human gastric cancer AGS cell line (wild-type p53; https://p53.iarc.fr/CellLines.aspx) was obtained from the Cancer Research Institute of Central South University. Cells were cultured in RPMI-1640 supplemented with 10% FBS (both Gibco; Thermo Fisher Scientific, Inc.) in a humidified incubator at 37°C with 5% CO_2_. AGS cells were stimulated with cisatracurium besilate [purity, >98%; ChemeGen (Shanghai) Biotechnology Co., Ltd.; 20, 40, 60 and 80 μg/ml] [9] for 48 h to assess the effects on AGS cell apoptosis. 5-Fluorouracil [5-FU; purity, >98%; ChemeGen (Shanghai) Biotechnology Co., Ltd.], which has been frequently used to treat advanced GC [13], was diluted to 5 μg/ml [14] for the determination of cell viability. TRAIL protein [purity, >95%; ACROBiosystems (Beijing) Biotechnology Co., Ltd., 100 ng/ml] [15] and pifithrin-α (p53 inhibitor; purity, >98%; Beyotime Institute of Biotechnology; 30 μg/ml) [16] were used to treat cells. Untreated cells were used as the control.

### Cell counting kit-8 (CCK-8) assay

For the detection of the cell inhibition rate and cell viability, AGS cells (5x10^3^/well) cultured in 96-well plates were treated with different concentrations of cisatracurium besilate (0, 20, 40, 60 or 80 μg/ml) or 5-FU (5 μg/ml) at 37°C for 24 or 48 h. Subsequently, 10 μl CCK-8 reagent (Dojindo Molecular Technologies, Inc.) was added to each well and incubated for another 4 h. Finally, the absorbance was measured at a wavelength of 450 nm using a microplate reader (BioTek Instruments, Inc.).

### Colony formation assay

AGS cells were seeded (3x10^4^ cells/well) into 6-well plates and treated with cisatracurium besilate (20, 40, 60 and 80 μg/ml). After 2 weeks of incubation at 37 °C, the formed colonies were fixed with 70% ethanol for 15 min and stained with 0.5% crystal violet solution (Sigma-Aldrich; Merck KGaA) for 30 min both at room temperature. Following washing with PBS, stained colonies (>50 cells) were photographed and counted by light microscopy (magnification, x100; Olympus Corporation).

### TUNEL

AGS cells were inoculated (3x10^4^/well) into a 24-well plate and cultured with cisatracurium besilate for 48 h. After washing with PBS, cells were fixed with 4% paraformaldehyde for 20 min at room temperature and then washed three times with PBS. Subsequently, cells were incubated with 75% ethanol at 4°C overnight. After washing with PBS, cell apoptosis was assessed using a TUNEL apoptosis kit [Yeasen (Shanghai) Biotechnology Co., Ltd.] according to the manufacturer’s protocol. TUNEL^+^ cells were visualized using a fluorescent microscope (magnification, x200) and quantified using ImageJ software (version 1.8; National Institutes of Health). The apoptosis rate (%) was calculated according to the following formula (Number of TUNEL^+^ cells/total number of cells) x100.

### Western blotting

Total proteins were isolated from treated AGS cells using lysis buffer (Beyotime Institute of Biotechnology) and quantified using a BCA Protein Assay Kit (Beyotime Institute of Biotechnology) according to the manufacturer’s protocol. Proteins (25 μg per lane) were separated by 10% SDS-PAGE and transferred onto PVDF membranes through semi-dry membrane transfer. After blocking with 5% skimmed milk overnight at 4°C, the membranes were incubated with primary antibodies at 4°C overnight. Subsequently, the membranes were incubated with a HRP-conjugated secondary antibody for 1.5 h at room temperature. The bands were visualized using a BeyoECL Plus kit (Beyotime Institute of Biotechnology). Protein expression was semi-quantified using ImageJ software with GAPDH as the loading control. The details of the antibodies are presented in Table 1.

**Table 1.** Antibodies used for Western blotting

Antibody	Dilution	Cat. no.	Manufacturer
Bcl2	1:1,000	ab32124	Abcam
Bax	1:1,000	ab32503	Abcam
Cleaved caspase-3	1:500	ab32042	Abcam
Cleaved PARP	1:10,000	ab32064	Abcam
p53	1:200	ab26	Abcam
PUMA	1:800	ab9643	Abcam
DR5	1:1,000	ab199357	Abcam
H3	1:1,000	ab1791	Abcam
GAPDH	1:10,000	ab181602	Abcam
IgG H&L (HRP)	1:10,000	ab6721	Abcam

### Statistical analysis

Data are presented as the mean ± SD. Statistical analyses were performed using GraphPad Prism software (version 8.0; GraphPad Software, Inc.). The unpaired Student’s t-test was used for comparisons between two groups, whereas one-way ANOVA followed by Tukey’s post hoc test was used to evaluate differences among multiple groups. P < 0.05 was considered to indicate a statistically significant difference. All experiments were performed three times.

## Results

To investigate the mechanism underlying the role of cisatracurium besilate in GC, AGS cell line was treated with cisatracurium besilate. And the result indicated that cisatracurium besilate treatment restrained the proliferation and promoted the apoptosis of AGS cells. In addition, cisatracurium besilate also promoted the expression of p53 and PUMA in AGS cells. Furthermore, TRAIL induced the apoptosis of AGS cells, which was aggravated by cisatracurium besilate treatment. However, pifithrin-α reversed the synergistic effects of cisatracurium besilate and TRAIL on the activities of AGS cells. Therefore, the present study suggested that cisatracurium besilate enhanced the TRAIL-induced apoptosis of GC cells via p53 signaling, and the synergistic effects of cisatracurium besilate and TRAIL increased the sensitivity of TRAIL to amplify the therapeutic efficacy in GC management. Moreover, the combined use of cisatracurium besilate and 5-FU drastically reduced cell viability, which indicated the possibility of drug combination.

### Cisatracurium besilate restrains the proliferation and apoptosis of AGS cells

**Figure 2.**
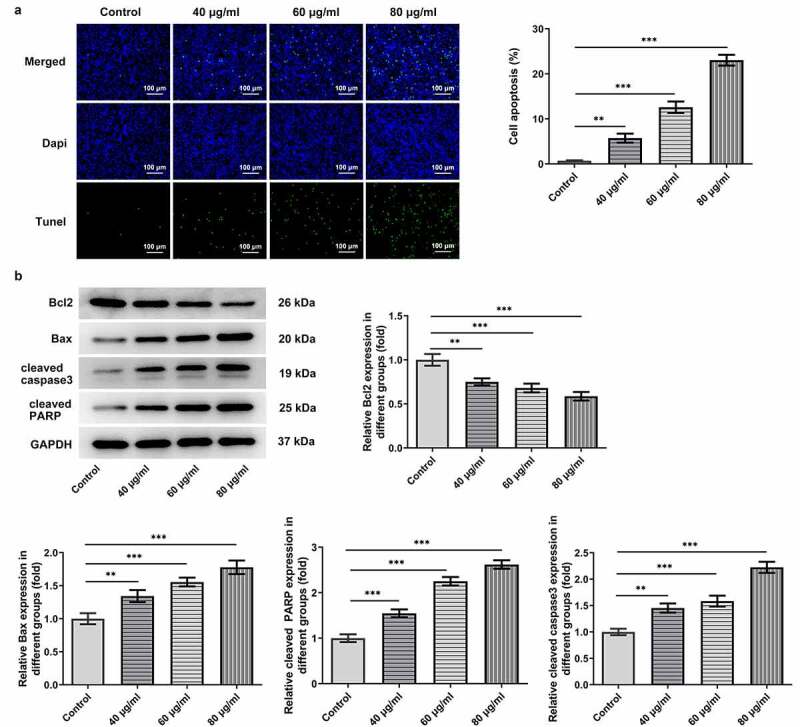
Cisatracurium besilate restrains the apoptosis of AGS cells. (a) TUNEL^+^ cells, cell apoptosis rate and (b) apoptosis-associated protein expression in cisatracurium besilate-treated AGS cells. **P < 0.01 and ***P < 0.001

The proliferation and apoptosis of AGS cells, which are considered as crucial markers of GC progression, were detected to assess the effects of cisatracurium besilate on GC. The inhibitory effect of cisatracurium besilate on AGS cells gradually increased in a dose-dependent manner (Figure 1b). Interestingly, the cell viability and colony formation abilities were both suppressed with increasing doses of cisatracurium besilate, as evidenced by decreased cell viability and reduced colony numbers in AGS cells following cisatracurium besilate exposure (Figure 1c–d). The effect of cisatracurium besilate on AGS cell apoptosis was assessed by performing TUNEL assays and Western blotting. The TUNEL assay results demonstrated that the fluorescence of apoptotic cells in the treatment group increased in a concentration-dependent manner, which indicated that AGS cell apoptosis was evidently upregulated by cisatracurium besilate (Figure 2a). The Western blotting results indicated that the protein expression levels of Bax, cleaved caspase-3 and cleaved poly (ADP-ribose) polymerase (PARP) were increased, whereas Bcl2 protein expression levels were decreased with increasing concentrations (Figure 2b). Taken together, these results demonstrated that cisatracurium besilate restrained the proliferation and promoted the apoptosis of AGS cells.

### Cisatracurium besilate enhances the apoptosis of AGS cells via p53

**Figure 3.**
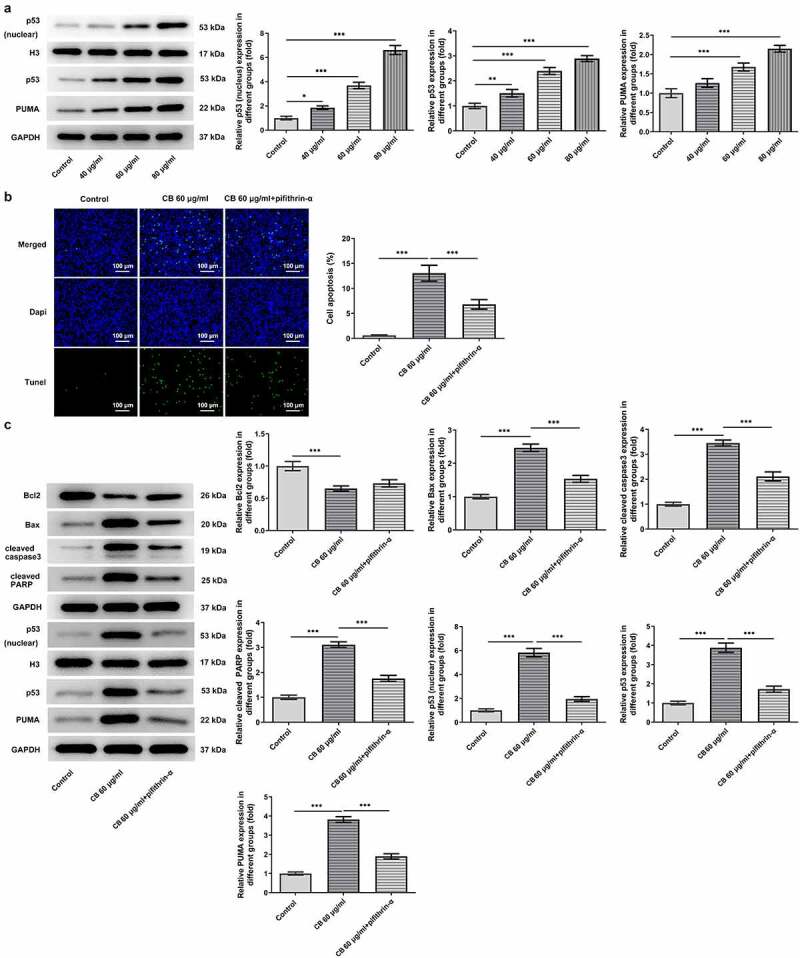
Cisatracurium besilate enhances the apoptosis of AGS cells via p53. (a) Expression of p53, p53 (nuclear) and PUMA in AGS cells treated with cisatracurium besilate. (b and c) Apoptosis and apoptosis-related protein expression in AGS cells treated with cisatracurium besilate and pifithrin-α. **P < 0.01 and ***P < 0.001. PUMA, p53 upregulated modulator of apoptosis

The predominant role of p53/PUMA in inducing apoptosis of cancer cells has previously been reported in numerous studies [17–19]. Thus, to investigate whether cisatracurium besilate inhibited the apoptosis of AGS cells via p53/PUMA, the expression levels of p53, PUMA, p53 (nuclear) and Histone H3 were measured. The Western blotting results revealed that cisatracurium besilate promoted the expression of p53 (nuclear), p53 and PUMA in AGS cells (Figure 3a). Subsequently, cisatracurium besilate-pretreated AGS cells were treated with 30 μM pifithrin-α for 2 h to observe alterations in cell apoptosis. Interestingly, pifithrin-α inhibited apoptosis (Figure 3b), enhanced the protein expression level of Bcl2 and suppressed the protein expression levels of Bax, cleaved caspase 3 and cleaved PARP. And pifithrin-α was found could also suppress the protein expression levels of p53, p53 (nuclear) and PUMA (Figure 3c). Thus, cisatracurium besilate enhanced the apoptosis of AGS cells via p53.

### Cisatracurium besilate enhances TRAIL-induced apoptosis of AGS cells

**Figure 4.**
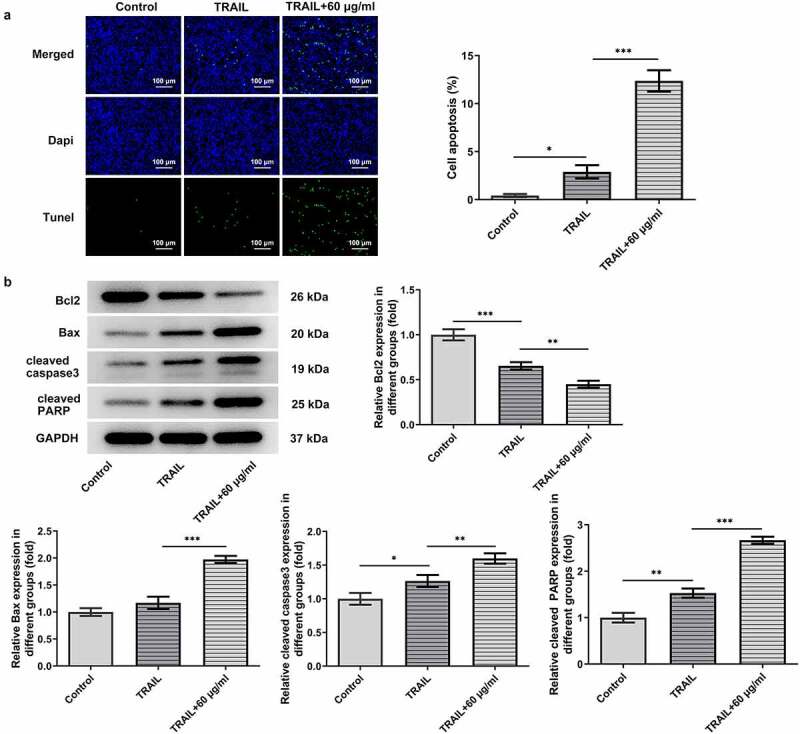
Cisatracurium besilate enhances the TRAIL-induced apoptosis of AGS cells. (a) Apoptosis and (b) apoptosis-related protein expression in AGS cells co-treated with cisatracurium besilate and TRAIL. *P < 0.05, **P < 0.01 and ***P < 0.001. TRAIL, TNF-related apoptosis-inducing ligand

As TRAIL can induce the apoptosis of cancer cells, the synergistic effects of cisatracurium besilate and TRAIL on the apoptosis of AGS cells were assessed. AGS cells were exposed to TRAIL and 60 μg/ml cisatracurium besilate. The TUNEL assay and Western blotting results indicated that co-administration of TRAIL and cisatracurium besilate markedly lowered the cell survival rate, downregulated the expression of Bcl2 and upregulated the expression of Bax, cleaved caspase-3 and cleaved PARP (Figure 4a–b). In conclusion, cisatracurium besilate enhanced TRAIL-induced apoptosis of AGS cells.

### Cisatracurium besilate enhances TRAIL-induced apoptosis of AGS cells via p53

**Figure 5.**
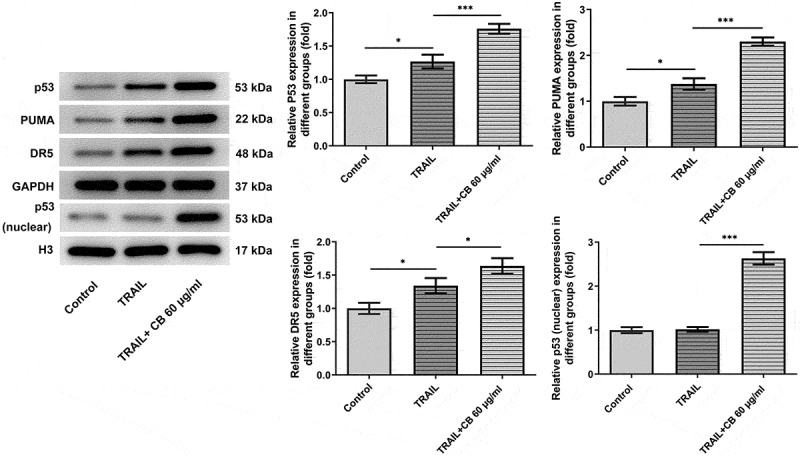
Cisatracurium besilate enhances the TRAIL-induced apoptosis of AGS cells via p53. Expression of p53, p53 (nuclear) PUMA and death receptors in AGS cells co-treated with cisatracurium besilate and TRAIL. *P < 0.05, **P < 0.01 and ***P < 0.001. TRAIL, TNF-related apoptosis-inducing ligand; PUMA, p53 upregulated modulator of apoptosis

**Figure 6.**
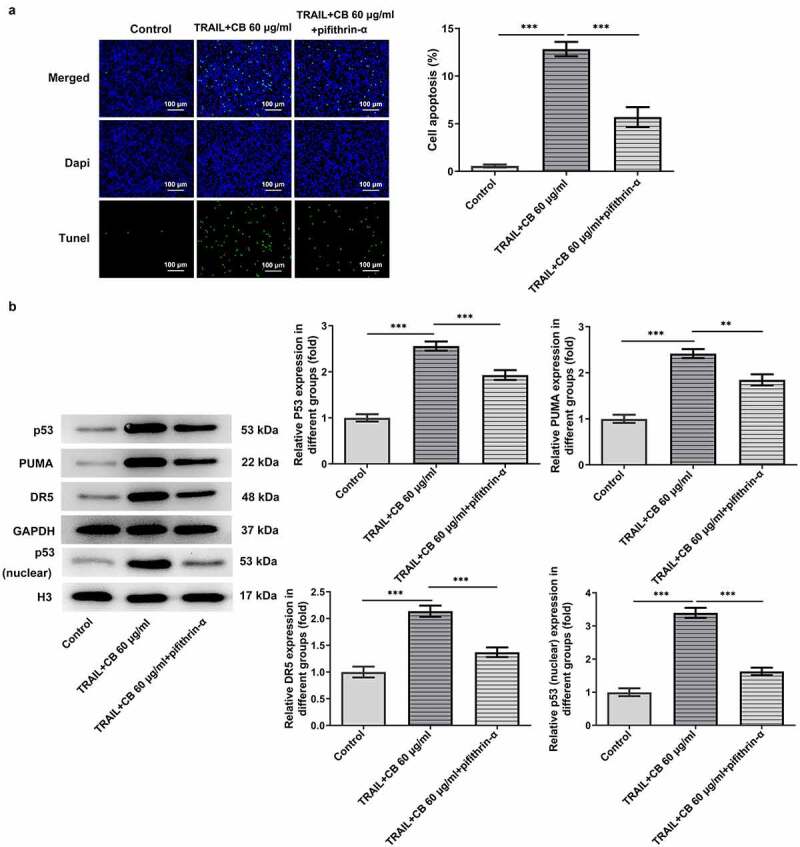
Cisatracurium besilate enhances the TRAIL-induced apoptosis of AGS cells via p53. (a) TUNEL^+^ cells, cell apoptosis rate and (b) expression of p53, p53 (nuclear) PUMA and death receptors in AGS cells treated with cisatracurium besilate. **P < 0.01 and ***P < 0.001. TRAIL, TNF-related apoptosis-inducing ligand; PUMA, p53 upregulated modulator of apoptosis

As suggested by previous findings, TRAIL can activate the apoptotic pathway by binding to associated death receptors death receptor (DR)4 and DR5 [20]. Thus, the present study investigated the synergistic effects of TRAIL and cisatracurium besilate on the expression of p53, p53 (nuclear), PUMA and DR5. It was discovered that TRAIL slightly promoted the expression of the expression of p53, p53 (nuclear), PUMA and DR5, whereas co-treatment with TRAIL and cisatracurium besilate drastically upregulated their expression (Figure 5). Subsequently, cells were incubated with pifithrin-α for another 2 h. Of note, treatment with pifithrin-α inhibited cell apoptosis, and simultaneously downregulated p53, p53 (nuclear), PUMA and DR5 expression compared that that in the TRAIL + 60 μg/ml cisatracurium besilate group (Figure 6a–b). These data indicated that cisatracurium besilate enhanced TRAIL-induced apoptosis of AGS cells via p53.

### The combined effect of cisatracurium besilate and 5-FU

**Figure 7.**
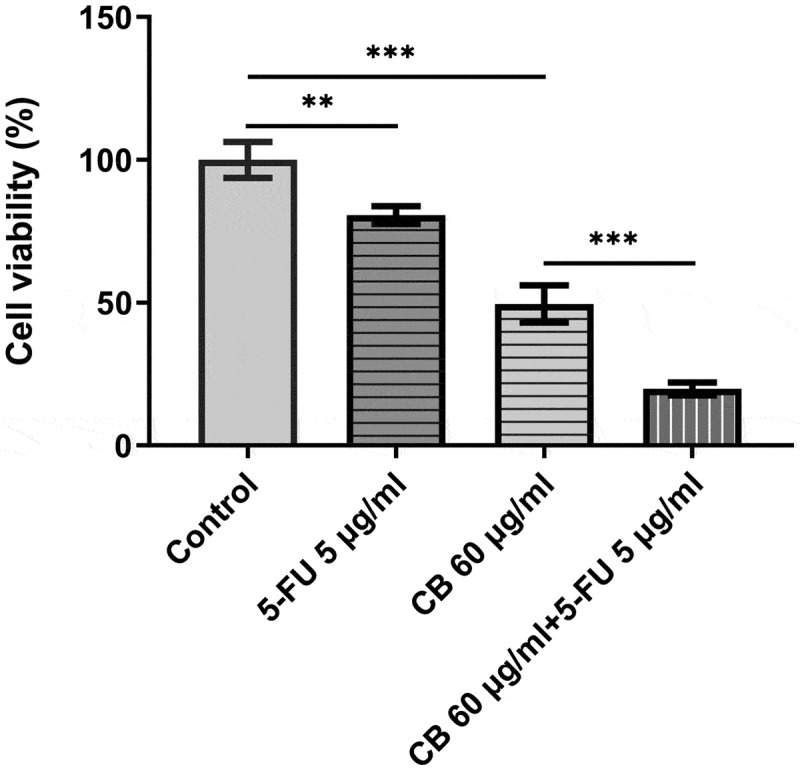
The combined effect of cisatracurium besilate and 5-FU. The viability of AGS cells was assessed using a CCK assay. **P < 0.01 and ***P < 0.001

5-FU has been used to treat advanced GC clinically. Therefore, their combined effect on GC cell viability are intrigued and of significance. The viability of AGS cells was assessed using a CCK assay. Following the treatment of 5-FU for 48 h, cell viability was significantly decreased. Novelty, the combined use of cisatracurium besilate and 5-FU drastically reduced cell viability, which is stronger than either single use (Figure 7).

## Discussion

Apoptosis is an ordered and orchestrated cellular process conducive to the regulation of cell proliferation in humans and animal species. The recently identified TRAIL, a unique type II transmembrane protein in the TNF family, is primarily expressed on a variety of cells that serve pivotal roles in inducing apoptosis [21]. As cancer cells are sensitive to TRAIL compared with normal cells, TRAIL was deemed to act as a powerful anticancer agent [22]. By means of associating with two agonistic TRAIL receptors, DR4 and DR5, the signaling molecules related to apoptosis are recruited to induce cell death [20]. A previous study indicated that encorafenib administration stimulates TRAIL-induced apoptosis of colorectal cancer cells via p53/PUMA signaling [18]; however, the precise molecular mechanism underlying the effect of cisatracurium besilate on TRAIL-induced apoptosis of AGS cells is not completely understood.

The failure of TRAIL to eliminate all the cancerous cells has constituted a major challenge for its clinical management of GC [23]. Drugs that can repair the apoptotic signal to a normal status can potently eradicate cancer cells, thus numerous scholars have paid attention to drugs that can induce the apoptosis of cancer cells [24]. Interestingly, studies aiming to identify novel drugs for cancer have indicated the important role of cisatracurium besilate in cancer treatment. For instance, cisatracurium besilate abated colorectal cancer cell migration and invasion, thereby suppressing the aggressiveness and metastasis of colorectal cancer [10]. Cisatracurium besilate also inhibited the malignant activities of breast cancer cells via targeting the expression of microRNA-3174 [25]. In the present study, increasing doses of cisatracurium besilate enhanced the cell inhibition rate, but decreased cell viability and colony formation abilities in a dose-dependent manner. Furthermore, the TUNEL assay and Western blotting results demonstrated that cell apoptosis was markedly induced by cisatracurium besilate, particularly at 80 μg/ml.

The p53 protein, which is encoded by the tumor suppressor gene tumor protein P53, is one of the most extensively investigated tumor suppressor proteins [24]. It is a critical participant in various physiological processes, including cell differentiation, cell cycle regulation and cell apoptosis [26,27]. Depletion of p53 is a common event in over half of the different types of human cancer [28,29]. 5-FU, which has been frequently used to treat advanced GC, has been shown to target nucleoli and cause some ribosomal proteins to induce p53-dependent cell apoptosis [13]. In the present study, it was hypothesized that cisatracurium besilate influenced the apoptosis of AGS cells by manipulating the expression of p53. Following treatment with cisatracurium besilate and pifithrin-α, the apoptosis of AGS cells was evidently decreased compared with that in cisatracurium besilate-treated cells, suggesting that cisatracurium besilate enhanced AGS cell apoptosis by regulating p53. Consistently, cisatracurium besilate triggered apoptosis in human colorectal cancer cells via the p53 intrinsic apoptotic pathway [12]. Subsequently, the synergistic effects of TRAIL and cisatracurium besilate on AGS cell apoptosis were assessed. As expected, the synergism of TRAIL and cisatracurium besilate achieved the optimal effects, as demonstrated by the finding that TRAIL and cisatracurium besilate jointly enhanced the cell apoptosis of AGS cells, and elevated the expression levels of p53, p53 (nuclear), PUMA and DR5. Furthermore, pifithrin-α reversed the suppressive effects of TRAIL and cisatracurium besilate on cell apoptosis, suggesting that cisatracurium besilate enhanced the TRAIL-induced apoptosis of gastric cancer cells via p53 signaling. In the absence of p53 will sharply promote tumor growth and metastasis in the body [30]. Only with a sufficiently comprehensive understanding of p53 signaling can it become a safe therapeutic target. However, the findings of the present study were only demonstrated in one type of p53-positive GC cell line, and p53 mutation has proven to play a non-negligible role in GC occurrence and prognosis [31,32]. Therefore, p53-negative GC cell lines, combined use with other drugs and *in vivo* experiments should be conducted in future studies to expand this study.

## Conclusion

In conclusion, the present study innovatively investigated the role of cisatracurium besilate in GC cells, particularly in GC cell apoptosis. The results demonstrated that cisatracurium besilate enhanced the TRAIL-induced apoptosis of GC cells via p53 signaling, suggesting that cisatracurium besilate increased the sensitivity of TRAIL to amplify the therapeutic efficacy of itself in GC management. In addition, the combination of cisatracurium besilate with 5-FU was found to drastically reduce GC cell viability, which indicated the possibility of drug combination.

## Data Availability

The datasets used and/or analyzed during the current study are available from the corresponding author on reasonable request.
